# Targeting the ZNF‐148/miR‐335/SOD2 signaling cascade triggers oxidative stress‐mediated pyroptosis and suppresses breast cancer progression

**DOI:** 10.1002/cam4.6673

**Published:** 2023-11-01

**Authors:** Yanmei Wang, Yansi Gong, Xuesha Li, Weizhao Long, Jiayu Zhang, Jiefang Wu, Yilong Dong

**Affiliations:** ^1^ Department of Breast Surgery First affiliated hospital of Kunming Medical University Kunming People's Republic of China; ^2^ School of Medicine Yunnan University Kunming People's Republic of China

**Keywords:** breast cancer, miR‐335, oxidative stress, pyroptosis, zinc finger protein 148

## Abstract

**Background:**

The implication of zinc finger protein 148 (ZNF‐148) in pathophysiology of most human cancers has been reported; however, the biological functions of ZNF‐148 in breast cancer remain unclear. This study sought to elucidate the potential molecular mechanism of ZNF‐148 on breast cancer pathology.

**Methods:**

ZNF148 expression was tested in breast cancer tissues and cells. Then, cells were transfected with ZNF‐148 overexpression or downregulation vector, and the cell proliferation, pyroptosis, apoptosis, and reactive oxygen species (ROS) production were analyzed by MTT, western blot, flow cytometry, and immunofluorescence staining, respectively. Tumor‐bearing nude mouse was used to evaluate tumorigenesis of ZNF‐148. Mechanisms underpinning ZNF‐148 were examined using bioinformatics and luciferase assays.

**Results:**

We found that ZNF‐148 was upregulated in breast cancer tissues and cell lines. Knockdown of ZNF‐148 suppressed malignant phenotypes, including cell proliferation, epithelial‐mesenchymal transition, and tumorigenesis in vitro and in vivo, while ZNF‐148 overexpression had the opposite effects. Further experiments showed that ZNF‐148 deficiency promoted ROS production and triggered both apoptotic and pyroptotic cell death, which were restored by cotreating cells with ROS scavengers. A luciferase reporter assay revealed that miR‐335 was the downstream target of ZNF‐148 and that overexpressed ZNF‐148 increased superoxide dismutase 2 (SOD2) expression by sponging miR‐335. In parallel, both miR‐335 downregulation and SOD2 overexpression abrogated the antitumor effects of ZNF‐148 deficiency on proliferation and pyroptosis in breast cancer cells.

**Conclusions:**

Our findings indicated that ZNF‐148 promotes breast cancer progression by triggering miR‐335/SOD2/ROS‐mediated pyroptotic cell death and aid the identification of potential therapeutic targets for breast cancer.

## 1 INTRODUCTION

As the most common malignancy among women, breast cancer is a multistep disease that develops by abnormal signal regulation in complex interactions. Therefore, the understanding of these interactions may contribute to explaining the potential mechanisms involved in breast cancer as well as the development of novel targeted therapies.

Zinc finger protein 148 (ZNF‐148), also known as ZBP89, is a Krüppel‐type zinc finger transcription factor that has been reported to be involved in many biological processes related to cancer, and it acts as a transcriptional regulator to activate or repress gene expression.^1^ In contrast, the role of ZNF148 in cancer is different. Previous studies have indicated that ZNF148 potentially acts as a tumor suppressor as reflected by its inhibitory effect on cell proliferation,^2^, ^3^ and other studies have suggested that ZNF148 may be an oncogene because it promotes tumor cell invasion and distal metastasis and is associated with poor prognosis.^4^, ^5^, ^6^ To date, studies on the role of ZNF‐148 in breast cancer have been limited, and the implications of its effects on breast cancer development are worthy of further investigation.

MicroRNAs (miRNAs) are a class of small noncoding RNAs that regulate posttranscriptional gene expression by binding to the 3′ untranslated regions (3′‐UTRs) of their target mRNAs, and they function as suppressors or promoters in various cancers,^7^ including breast cancer.^8^, ^9^, ^10^ Accumulating studies have shown that microRNA‐335 (miR‐335) is closely correlated with the development of multiple cancers, such as ovarian cancer,^11^ cervical cancer,^12^ lung cancer,^13^ and gastric cancer.^14^ Additionally, our previous study showed that miR‐335 acts as a suppressor to hinder the progression of breast cancer.^15^ In addition, Gao et al.^16^ reported that miR‐335 is a downstream target of ZNF‐148. Therefore, it is reasonable to speculate that there is a ZNF‐148/miR‐335 axis that regulates the cell pathogenesis of breast cancer.

Pyroptosis is an emerging form of programmed cell death and has been confirmed as an important effector to inhibit the malignant phenotype of breast cancer.^17^ Activation of caspase‐1 within the nucleotide‐binding oligomerization domain (NOD)‐like receptor containing pyrin domain 3 (NLRP3) inflammasome leads to the processing of interleukin‐1β (IL‐1β) and IL‐18 precursors into mature IL‐1β and IL‐18, which triggers pyroptosis.^18^ Although the mechanisms of NLRP3 activation are not completely understood, increasing evidence supports that reactive oxygen species (ROS) may serve as a “kindling” factor to activate NLRP3 inflammasomes as well as “bonfire” factors, resulting in pathological processes.^19^ Thus, superoxide dismutase 2 (SOD2) may play a role in regulating pyroptosis because it is a mitochondrial antioxidative enzyme involved in ROS elimination.^20^ It has been reported that high expression levels of SOD2 are accompanied by low expression of pyroptosis‐related proteins.^21^ Of note, SOD2 is suppressed by miR‐335.^22^ Importantly, Liu et al.^23^ reported that SOD2 silencing induces excessive oxidative damage and ROS production, resulting in the initiation of pyroptotic cell death in non‐small cell lung cancer, and they also demonstrated that miR‐335 targets the 3′‐UTRs of SOD2 mRNA. Based on these findings, we hypothesized that miR‐335 may be the “bridge” to combine ZNF‐148 and SOD2 and that ZNF‐148 may be involved in breast cancer progression by targeting miR‐335/SOD2 signaling. Further, we hypothesized that oxidative stress and pyroptosis may also be crucial for breast cancer pathogenesis mediated by ZNF‐148.

In the present study, we identified a ZNF‐148/miR‐335/SOD2 axis in breast cancer cells, and we demonstrated that ZNF‐148 represses miR‐335 and then increases SOD2 expression, consequently decreasing pyroptosis in a ROS‐dependent manner. This study may offer novel mechanistic insights into the excitatory signature of ZNF148 in the development of breast cancer.

## 2 MATERIALS AND METHODS

### 2.1 Clinical tissue collection and analysis

The 35 paired breast cancer tissues and the corresponding adjacent normal breast tissues were collected from female patients at the First Affiliated Hospital of Kunming Medical University from July 2020 to July 2021. After collection, the tissues were immediately stored at −80°C for further utilization. All participants signed informed consent forms, and the associated clinical experiments were approved by the Ethical Committee of the First Affiliated Hospital of Kunming Medical University.

### 2.2 Cell culture, vector transfection, and treatments

Breast cancer cells (T47D and MDA‐MB‐468) and normal human breast epithelial cells (MCF‐10A) were obtained from the Kunming Cell Bank of Chinese Academy of Sciences (Kunming, China). The breast cancer cell lines were cultured in Dulbecco's Modified Eagle Medium (DMEM; Invitrogen Corporation, Cat#11965118), and the normal MCF‐10A cells were maintained in Roswell Park Memorial Institute‐1640 (RPMI‐1640) medium (Invitrogen, Cat#11875119). All culture media were supplemented with 10% fetal bovine serum (FBS; Invitrogen, Cat#10099141C), 100 U/mL penicillin, and 100 μg/mL streptomycin (Invitrogen, 15140122). Cells were cultured in an incubator with 5% CO_2_ at 37°C, and they were utilized for experiments when the cell confluency reached approximately 70% at passages 3–6. The ZNF‐148 overexpression vector, ZNF‐148 downregulation vector, miR‐335 mimic, miR‐335 inhibitor, and SOD2 overexpression vectors (all from Sangon Biotech) were delivered into cells using Lipofectamine 3000 reagent (Invitrogen, Cat#L3000015) based on the protocols provided by the manufacturer. In addition, cells were exposed to the ROS scavengers, N‐acetylcysteine (NAC; Sigma–Aldrich, Cat#A0737), and alpha‐lipoic acid (ALA; Sigma–Aldrich, Cat#1368201), for 24 h under standard culture conditions.

### 2.3 Quantitative real‐time PCR (qRT‐PCR)

The clinical tissues and associated cell lines were washed and prepared, and TRIzol reagent (Invitrogen, Cat#15596026CN) was used for total RNA extraction, which was examined by agarose electrophoresis. RNA was reverse transcribed into cDNA using the Quantiscript reverse transcription kit (QIAGENG, Cat#205313), and SYBR qPCR Mix (QIAGENG, Cat#330502) was employed to quantify the mRNA levels of target genes. Gene expression levels were quantified by normalizing the Ct values of target genes to those of the reference gene (GAPDH) with the ΔΔCt method. The following primer sequences for target genes (Sangon Biotech) were used for qRT‐PCR: ZNF‐148F, 5′‐TGA TGA TGC CAT GCA GTT TT‐3; ZNF‐148R, 5′‐TCC CTG CTG TTG TTA CTT GCT‐3′; miR‐335F, 5′‐TCA AGA GCA ATA ACG AAA AAT GT‐3′; miR‐335R, 5′‐GCT GTC AAC GAT ACG CTA CGT‐3′; SOD2F, 5′‐CCC AGA TAG CTC TTC AGC CTG CAC T‐3′; SOD2R, 5′‐TAA GCG TGC TCC CAC ACA TCA ATC C‐3′; U6F, 5′‐CTC GCT TCG GCA GCA CA‐3′; U6R, 5′‐AAC GCT TCA CGA ATT TGC GT‐3′; GAPDHF, 5′‐GGT GAA GGT CGG AGT CAA CG‐3′; and GAPDHR, 5′‐CAA AGT TGT CAT GGA‐3′.

### 2.4 Western blot analysis

Total proteins were extracted from the clinical tissues and cells using RIPA lysis buffer (Beyotime), and the protein concentrations were determined using a BCA protein assay kit (ThermoFisher, Cat#23227). SDS–PAGE was then performed to separate the proteins according to their molecular weight, and the target protein bands were transferred onto PVDF membranes. After blocking the membranes with 5% nonfat milk, they were incubated with the following primary antibodies at 4°C overnight: ZNF‐148 (1:1500, ab69933), N‐cadherin (1:1000, ab76011), vimentin (1:2000, ab16700), Bax (1:2000, ab32503), Bcl‐2 (1:1500, ab32124), NLRP3 (1:2000, ab263899), ASC (1:2000, ab151700), IL‐1β (1:1500, ab216995), IL‐18 (1:2000, ab207324), and GAPDH (1:2000, ab128915). The membranes were then incubated with the secondary antibody (1:3000, ab205718) for 1 h at room temperature. Finally, an ECL kit (ThermoFisher, Cat#32134) was employed to visualize the protein bands, which were quantified using ImageJ software. All antibodies were purchased from Abcam.

### 2.5 Evaluation of cell proliferation

Cells were seeded into 96‐well plates at a density of 5 × 10^4^ cells/well in an incubator with standard culture conditions for 0, 24, 48, and 72 h, and the MTT Assay kit (Beyotime, Cat#C0009S) was used to evaluate cell proliferation. Briefly, cells were incubated with the MTT reaction solution (10 μL per well) for 4 h at 37°C. After diluting with DMSO, the absorbance at 578 nm was measured using a scanning microplate reader (Tecan).

### 2.6 Quantification of ROS production

Reactive oxygen species production in breast cancer cells was measured using DCFH‐DA probes (Sigma–Aldrich, Cat#35845) and dihydroethidium (DHE) (Sigma–Aldrich, Cat#37291) staining assays. For the DCFH‐DA assay, cells were incubated with DCFH‐DA probes for 20 min at 37°C, and the ROS levels were quantified using flow cytometry (BD Biosciences). For the DHE staining assay, cells were incubated with DHE staining solution for 60 min at 37°C. A fluorescence microscope (Leica) was then used to observe and acquire images, and the fluorescence intensity was used to evaluate ROS levels in the cells.

### 2.7 Flow cytometry analysis of cell apoptosis

Apoptotic cell death was quantified by flow cytometry using an Annexin V‐FITC kit (BD Pharmingen, Cat#556570) according to the manufacturer's protocol. Briefly, cells were sequentially treated with Annexin V‐FITC and PI staining solution for 30 min at 37°C without light exposure. A FACSCalibur machine (BD Bioscience) was employed to measure the apoptotic Annexin V‐FITC‐ and PI‐positive cells.

### 2.8 Dual‐luciferase reporter gene system assay

After acquiring the targeting sites of miR‐335 with ZNF‐148 and SOD2 mRNA from previous literature, the binding sites in ZNF‐148 and SOD2 mRNA were mutated, and the wild‐type (Wt) and mutant‐type (Mut) sequences were cloned into luciferase reporters (Sangon Biotech). The above vectors were then cotransfected with miR‐335 mimic into breast cancer cells using Lipofectamine reagent (Invitrogen) according to the manufacturer's instructions. At 48 h posttransfection, a luciferase reporter system (Promega, Cat#E1910) was utilized to detect relative luciferase activities.

### 2.9 Establishment of tumor‐bearing mouse models

Eight‐week‐old nude mice were purchased from Beijing Vital River Laboratory Animal Technology and were fed in specific‐pathogen‐free (SPF) conditions with free access to food and water. Cells were suspended in PBS buffer and subcutaneously injected into the back flank of each mouse at a concentration of 5 × 10^6^ per mouse. The mice were monitored every 7 days and were sacrificed at 56 days postinjection. The tumors were obtained, weighed, and photographed to evaluate tumorigenesis of the injected cells. All the animal experiments were approved by the Ethics Committee of the First Affiliated Hospital of Kunming Medical University.

### 2.10 Statistical analysis

All data are presented as the mean ± standard deviation (SD) and were analyzed by SPSS 18.0 software (SPSS Inc.). Statistical differences between two groups were determined using Student's *t* test, and one‐way ANOVA was conducted among multiple groups. Each experiment was repeated at least three times to avoid systemic and random errors/bias, and *p* < 0.05 was regarded as statistically significant. The PCR and western blot data were normalized using housekeeping genes and setting the value of the control group to 1 to compare the changes of target genes and proteins in groups. All images were drawn by GraphPad Prism 8.0 software (GraphPad).

## 3 RESULTS

### 3.1 ZNF‐148 facilitates the development of breast cancer in vitro and in vivo

**FIGURE 1:**
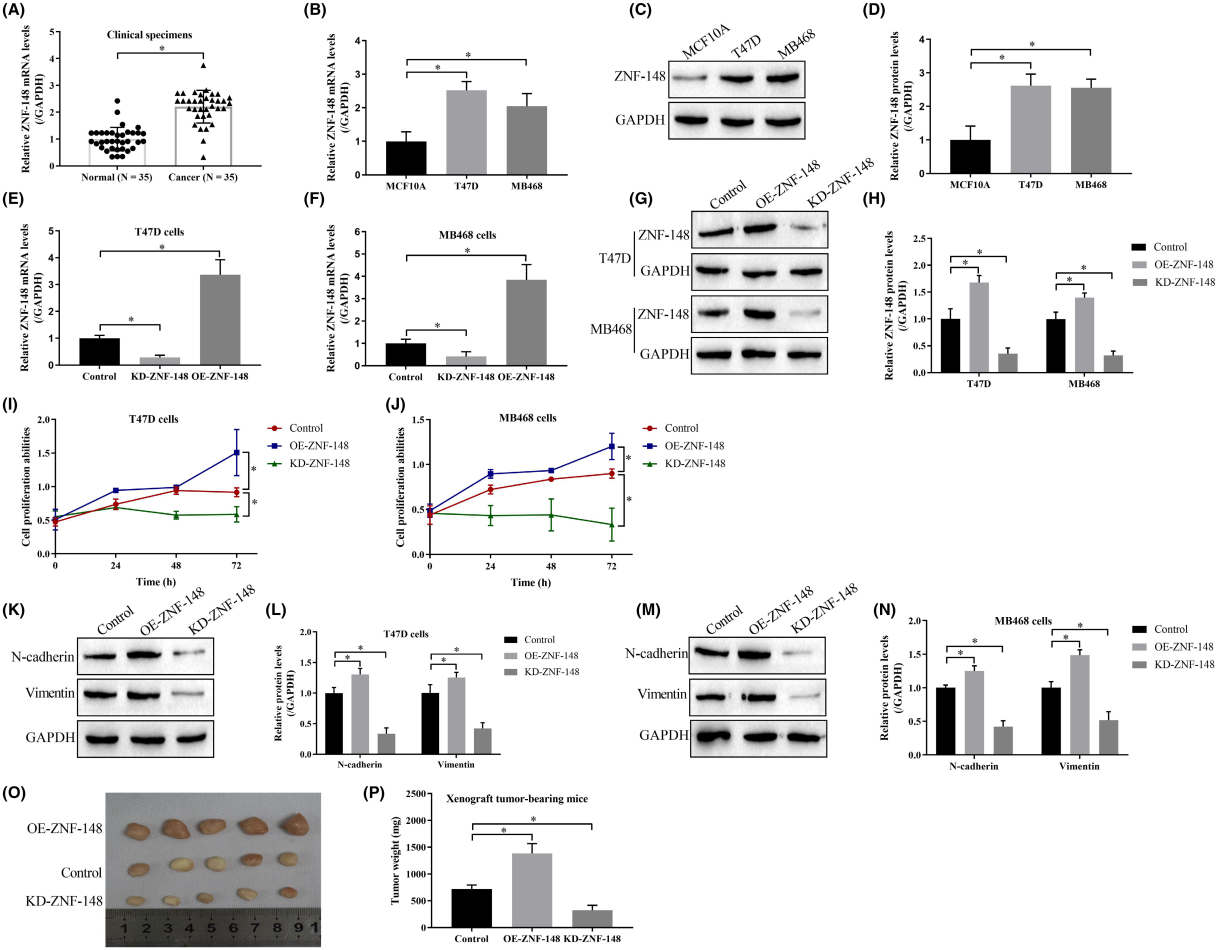
ZNF‐148 acts as an oncogene in breast cancer. qRT‐PCR was performed to determine ZNF148 mRNA levels in breast cancer tissues (A) and cells (B). ZNF‐148 proteins in breast cancer cells were measured by using Western blot analysis (C, D). ZNF‐148 was overexpressed and silenced in breast cancer cells (E–H). An MTT assay was conducted to measure cell proliferation (I, J). EMT markers were detected by Western blot analysis (O, P). Tumors were obtained and weighed to evaluate breast cancer cell growth in vivo (K–N). Each experiment was repeated at least three times (*p < 0.05). OE means the gene expression has been upregulated; KD means the gene expression has been downregulated.

Breast cancer tissues (*n* = 35) and their paired adjacent normal tissues (*n* = 35) were collected for further analysis, and qRT‐PCR analysis demonstrated that ZNF‐148 was upregulated in cancer tissues compared to normal tissues (Figure 1A). Additionally, further cellular results validated that ZNF‐148 was enriched in breast cancer cell lines (T47D and MB468) but not in normal cells (Figure 1B–D). ZNF‐148 overexpression and downregulation vectors were transfected into breast cancer cells (Figure 1E–H), and the associated malignant phenotypes were examined. Specifically, the MTT assay results showed that knockdown of ZNF‐148 inhibited cell proliferation in breast cancer cells, while ZNF‐148 overexpression had the opposite effect (Figure 1I,J). In addition, knockdown of ZNF‐148 downregulated the expression of N‐cadherin and vimentin (Figure 1K–N), which are involved in epithelial‐mesenchymal transition (EMT) in breast cancer cells. Finally, ZNF‐148‐overexpressing and ZNF‐148‐deficient T47D cells were used to establish xenograft tumor‐bearing mouse models, which demonstrated that ZNF‐148 promoted tumorigenesis in vivo (Figure 1O,P).

### 3.2 Knockdown of ZNF148 triggers apoptotic and pyroptotic cell death as well as promotes oxidative stress in breast cancer cells

**FIGURE 2:**
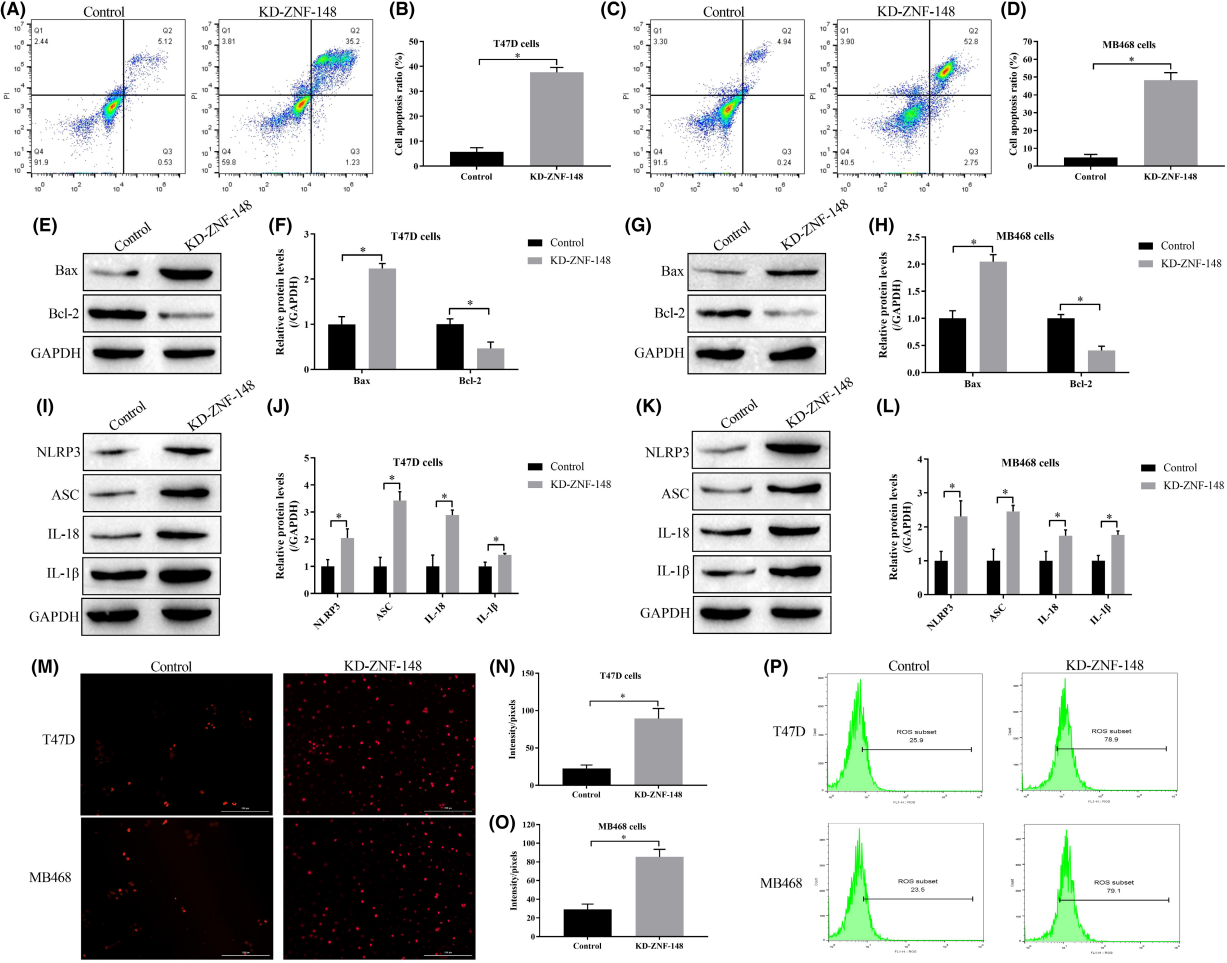
Knockdown of ZNF148 regulates cell apoptosis, pyroptosis, and oxidative stress in breast cancer cells. Cells were stained with Annexin V‐FITC and PI, and FCM was performed to detect the cell apoptosis ratio (A–D). Western blot analysis was performed to measure the expression levels of apoptosis‐ and pyroptosis‐associated proteins in breast cancer cells (E–L). ROS were quantified using the DCFH‐DA assay (M–O) and DHE staining assay (P). Each experiment was repeated at least three times (*p < 0.05). KD means the gene expression has been downregulated.

To determine the effect of ZNF148 on cell death, an Annexin V‐FITC/PI double staining assay was performed to evaluate cell apoptosis. Knockdown of ZNF‐148 significantly increased the cell apoptosis ratio in breast cancer cells (Figure 2A–D). Consistently, Western blot analysis demonstrated that ZNF‐148 deficiency upregulated Bax (proapoptotic molecule) but downregulated Bcl‐2 (antiapoptotic molecule) in cells (Figure 2E–H). In addition, ZNF‐148 regulated cell pyroptosis. Figure 2I–L shows that ZNF‐148 deficiency upregulated NLRP3, ASC, IL‐1β, and IL‐18 to promote pyroptotic cell death in the cells. Specifically, the cells were stained with DCFH‐DA probes and DHE, and we noticed that silencing ZNF‐148 promoted the generation of reactive oxygen species (ROS) in the cells (Figure 2M‐P).

### 3.3 ZNF‐148 deficiency promotes cell apoptosis and pyroptosis by triggering oxidative damage

**FIGURE 3:**
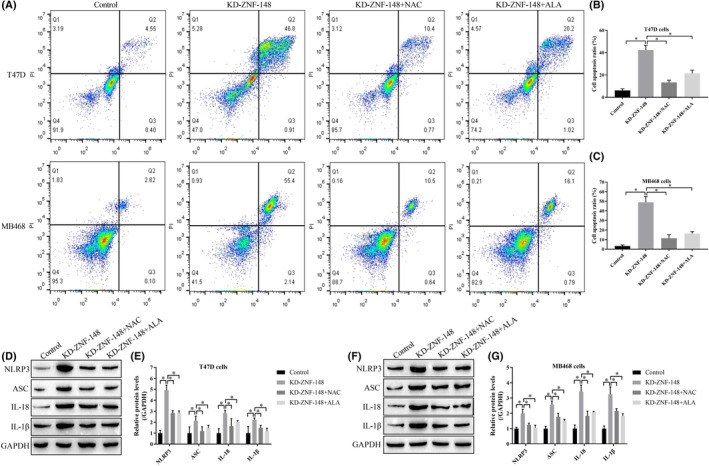
Silencing ZNF‐148 induces cell apoptosis and pyroptosis in an oxidative stress‐dependent manner. An Annexin V‐FITC/PI double staining assay was performed to determine cell apoptosis (A–C). Western blot analysis was used to determine the expression status of pyroptosis‐associated proteins (D–G). Each experiment was repeated at least three times (*p < 0.05). KD means the gene expression has been downregulated.

Given that oxidative stress was closely associated with both cell apoptosis and pyroptosis and that ZNF‐148 regulated ROS production in breast cancer cells, we hypothesized that ZNF‐148 may regulate cell death in an oxidative stress‐dependent manner. To validate this hypothesis, breast cancer cells were pretransfected with ZNF‐148 downregulation vectors and subsequently treated with ROS scavengers (NAC and ALA). Both NAC and ALA decreased the cell apoptosis ratio in ZNF‐148‐deficient cells (Figure 3A–C). Western blot analysis demonstrated that knockdown of ZNF‐148 increased the expression levels of pyroptosis‐associated biomarkers (NLRP3, ASC, IL‐1β, and IL‐18) in breast cancer cells, which was reversed by cotreating cells with NAC and ALA (Figure 3D–G). Together, these results suggested that silencing ZNF‐148 promotes cell apoptosis and pyroptosis by triggering oxidative stress.

### 3.4 ZNF‐148 regulates SOD2 levels by targeting miR‐335

**FIGURE 4:**
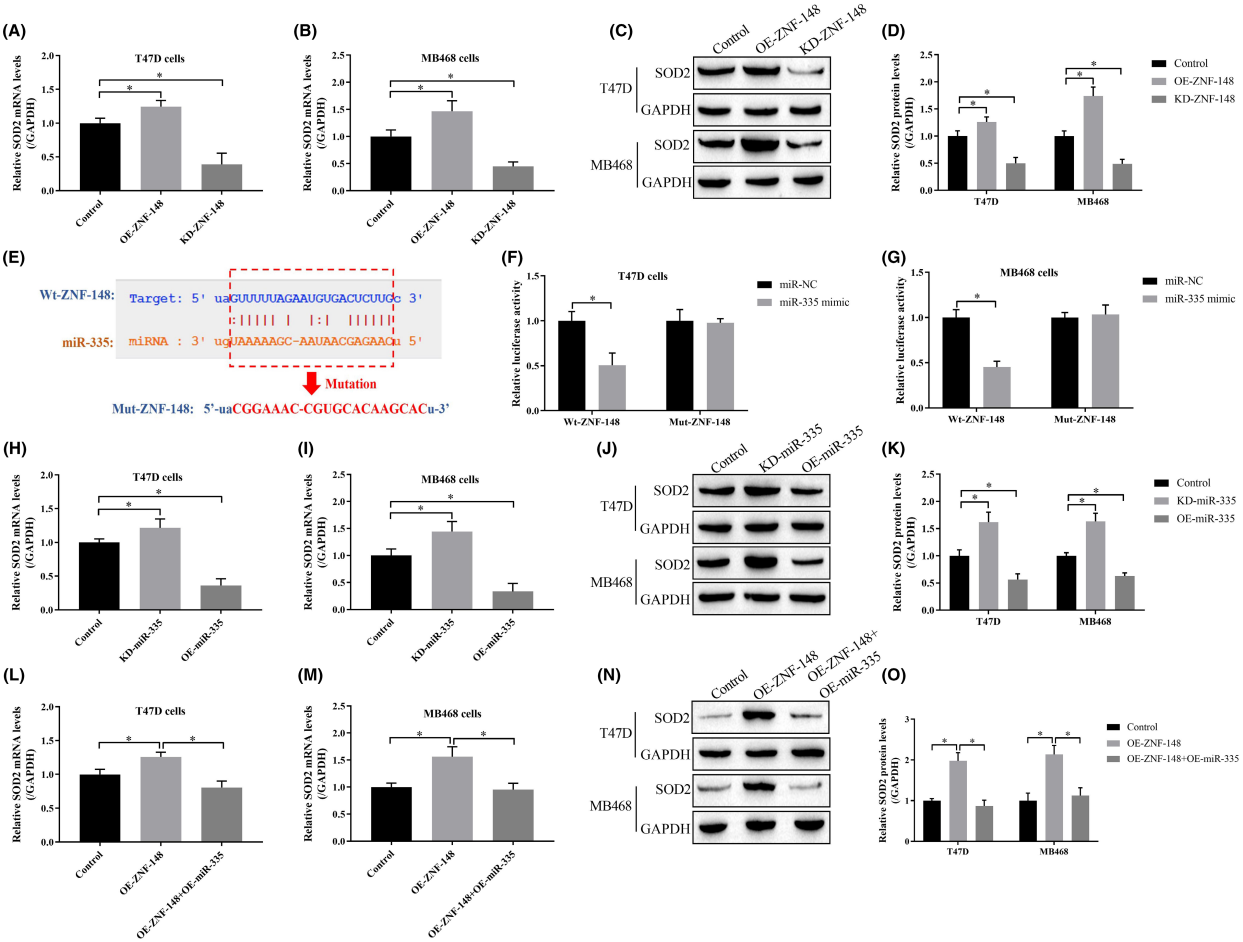
Regulatory mechanisms of ZNF‐148, miR‐335, and SOD2 in breast cancer cells. ZNF‐148 positively regulated SOD2 expression in breast cancer cells as determined by qRT‐qPCR and Western blot analyses (A–D). The targeting sites in miR‐335 and ZNF‐148 were predicted by gene detection software (E), and validated miR‐335 is a target of ZNF‐148 using the dual‐luciferase reporter assay (F, G). miR‐335 suppressed SOD2 expression at both the transcriptional and translational levels (H–K). ZNF‐148 upregulated SOD2 expression by sequestering miR‐335 (L–O). Each experiment was repeated at least three times (*p < 0.05). OE means the gene expression has been upregulated; KD means the gene expression has been downregulated.

To test the hypothesis that miR‐335 may be the “bridge” to link ZNF‐148 and SOD2, we investigated the potential associations between them. Overexpression and downregulation of ZNF‐148 in breast cancer cells demonstrated that ZNF‐148 positively regulated SOD2 (Figure 4A–D). Next, PicTar (www.pictar.org), TargetScan (www.targetscan.org) and miRanda (www.microrna.org) prediction software suggested that miR‐335 was downstream of ZNF‐148 (Figure 4E), and a dual‐luciferase reporter assay demonstrated that miR‐335 is a target of ZNF‐148 in breast cancer cells (Figure 4F,G). Furthermore, miR‐335 negatively regulated SOD2 expression in breast cancer cells (Figure 4H–K). Finally, we demonstrated that the miR‐335 mimic abrogated the promoting effects of ZNF‐148 overexpression in SOD2 in breast cancer cells (Figure 4L–O), suggesting that miR‐335 is a link between ZNF‐148 and SOD2.

### 3.5 Knockdown of ZNF148 promotes apoptotic and pyroptotic cell death by modulating the miR‐335/SOD2 axis

**FIGURE 5:**
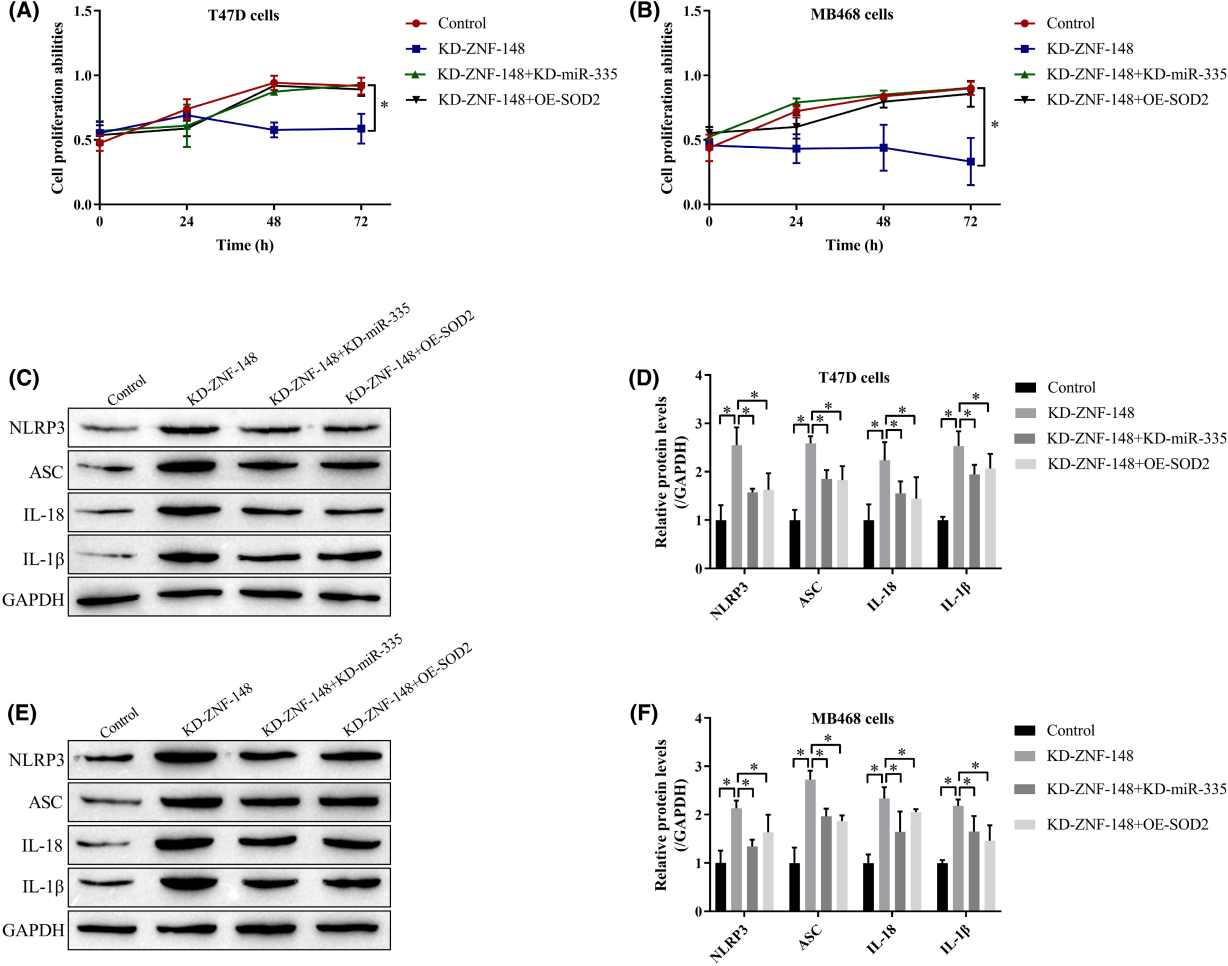
ZNF‐148 deficiency suppresses cell viability and promotes pyroptotic cell death by modulating the miR‐335/SOD2 axis. An MTT assay was performed to determine cell proliferation (A, B). Western blot analysis was used to quantify the protein levels of pyroptosis‐associated proteins in breast cancer cells (C–F). Each experiment was performed at least three times (*p < 0.05). OE means the gene expression has been upregulated; KD means the gene expression has been downregulated.

Because both miR‐335 and SOD2 are involved in regulating pyroptosis, we next explored whether ZNF‐148 modulates this progression through the miR‐335/SOD2 axis by transfecting the ZNF‐148 silencing vector, miR‐335 inhibitor, and SOD2 overexpression vector into breast cancer cells. The MTT assay showed that silencing ZNF‐148 inhibited cell proliferation, and this effect was reversed by the miR‐335 inhibitor and SOD2 overexpression (Figure 5A,B). In addition, knockdown of ZNF‐148 upregulated NLRP3, ASC, IL‐1β, and IL‐18 to promote cell pyroptosis in cells, and these effects were abrogated by suppressing miR‐335 and upregulating SOD2 (Figure 5C–F).

## 4 DISCUSSION

ZNF148 is universally expressed at low levels in most tissues,^24^ and its upregulation has been reported to be closely related to the deterioration of some tumors.^4^, ^5^, ^6^ In the present study, ZNF‐148 was overexpressed in breast cancer tissues and cell lines, which agreed with previous evidence,^25^ and further, gain‐ and loss‐of‐function experiments showed that ZNF‐148 positively regulated malignant phenotypes, including cell proliferation, epithelial‐mesenchymal transition (EMT), and in vivo tumorigenesis in breast cancer. Thus, these findings demonstrated that ZNF‐148 acts as a tumor activator in breast cancer to promote tumor progression.

To further investigate the molecular mechanism underlying ZNF‐148‐mediated tumorigenesis, apoptosis was evaluated because the decrease in programmed cell death is an important cause of breast cancer.^26^ The present study demonstrated that the expression of the proapoptotic protein, Bax, increased but that the expression of the antiapoptotic protein, Bcl‐2, decreased when ZNF‐148 was knocked down in breast cancer cells. Corresponding to the changes in Bax and Bcl‐2, the apoptosis rate was increased in ZNF‐148‐deficient cells. In contrast, Bai et al.^3^, ^27^ reported that high ZNF‐148 expression, not low ZNF‐148 expression, benefits apoptotic cell death in gastrointestinal cancer. Based on these contradictory findings, we speculated that ZNF‐148 has a double‐edged effect of promoting cancer cell proliferation and inhibiting proliferation depending on its different phenotypes. Recent data have revealed that ZNF‐148 has ZNF148^FL^ and ZNF148^ΔN^ splicing isoforms; ZNF148^FL^ decreases apoptotic cell death, while ZNF148^ΔN^ has the opposite effect.^28^ These findings partly explain why ZNF‐148 silencing promoted breast cancer cell apoptosis in the present study and why ZNF‐148 overexpression mediated apoptotic cell death in the study by Bai et al.,^3^, ^27^ indicating different ZNF‐148 splicing isoforms may exist in breast cancer, gastrointestinal tumors, or other tumors. Moreover, Bai et al. also found that ZNF‐148 promotes apoptosis through wild‐type p53 signaling, but another study has reported that the mutation of p53 in breast cancer is as high as 60%–80%.^29^ Thus, these findings suggest that ZNF‐148 may not function with mutated p53 to attenuate its effect on apoptosis in breast cancer cells. Further research is necessary to explore these differences.

Knockdown of ZNF148 increased the proposed pyroptosis‐associated biomarkers in breast cancer cells, indicating pyroptotic cell death. To the best of our knowledge, the regulatory effects of ZNF‐148 on cell pyroptosis have not been mentioned in existing publications. We also investigated the changes in the production of ROS because ROS are critical mediators of pyroptosis.^30^ In addition to increased pyroptosis, ROS production was significantly increased in ZNF‐148‐deficient cells, but the effects of ZNF‐148 in promoting pyroptosis were diminished by ROS scavenging. At the same time, we noticed that ROS scavengers could not completely eliminate pyroptosis induced by ZNF‐148 knockdown. Jiang and colleagues found that RNAi of ZNF‐148 increased the expression of tumor necrosis factor‐alpha (TNF‐α),^31^ the cytokine which had a priming effect on the activation of NLRP3 inflammasome.^32^ Maybe this is why scavenge of ROS did not completely block pyroptosis in the absence of ZNF‐148 since the pyrolytic cell death mediated by TNF‐α still work. Although the crosstalk between ROS and TNF‐α on pyroptosis needs more researches, our findings also supported that ZNF148 regulates pyroptosis via an oxidative stress mechanism.

SOD2 removes ROS and participates in oxidative stress,^20^ which may be involved in pyroptosis. The present study found that ZNF‐148 positively correlated with SOD2, suggesting a role in the pathogenesis mediated by ZNF‐148. Moreover, our results showed that the expression of SOD2 was suppressed by miR‐335, and bioinformatics analysis and luciferase reporter assays identified miR‐335 as the downstream target of ZNF‐148. SOD2 has been demonstrated to be targeted by miR‐335,^23^ supporting our hypothesis that miR‐335 serves as a “bridge” to combine ZNF‐148 and SOD2 in breast cancer cells, and we demonstrated that a ZNF‐148/miR‐335/SOD2 pathway exists in breast cancer. Mechanistically, ZNF‐148 positively regulated SOD2 through miR‐335 in a competing endogenous RNA (ceRNA)‐dependent manner as proposed by Salmena et al.^33^ Upregulation of ZNF‐148 occupied miR‐335 in breast cancer cells, and the remaining miR‐335 was not sufficient to target the 3′‐UTR of SOD2 mRNA, resulting in SOD2 upregulation.

In addition, we investigated the role of the ZNF‐148/miR‐335/SOD2 pathway in regulating breast cancer pathogenesis and found that knockdown of ZNF‐148 suppressed cell viability by downregulating SOD2 by sequestering miR‐335. Our data were partially supported by previous studies showing that miR‐335 acts as a tumor suppressor^15^ and that SOD2 acts as an oncogene in breast cancer.^34^ Along with the suppression of breast cancer malignancy in ZNF‐148‐deficient cells, the present data showed that ZNF‐148 silencing upregulated pyroptosis‐associated biomarkers (NLRP3, ASC, IL‐1β, and IL‐18) in breast cancer cells, but both miR‐335 knockdown and SOD2 overexpression abrogated the promoting effects of ZNF‐148 deficiency on breast cancer cell pyroptosis. The above findings confirmed that ZNF‐148 modulates cell pyroptosis in breast cancer cells via the miR‐335/SOD2 axis.

Taken together, we concluded that ZNF‐148 acts as an oncogene to facilitate breast cancer pathogenesis, and knockdown of ZNF‐148 triggers oxidative stress‐mediated pyroptotic cell death in breast cancer by downregulating SOD2 in a miR‐335‐dependent manner. The present study identified a ZNF‐148/miR‐335/SOD2 feedback loop that regulates breast cancer development, thereby suggesting ideal targets for breast cancer treatment in the clinic.

## AUTHOR CONTRIBUTIONS

**Yanmei Wang:** Data curation (equal); formal analysis (equal); funding acquisition (equal); investigation (equal); methodology (equal); writing – original draft (equal). **Yansi Gong:** Investigation (equal); methodology (equal). **Xuesha Li:** Investigation (equal); methodology (equal). **Weizhao Long:** Investigation (equal); methodology (equal). **Jiayu Zhang:** Investigation (equal); methodology (equal). **Jiefang Wu:** Investigation (equal); methodology (equal). **Yilong Dong:** Conceptualization (equal); project administration (lead); supervision (lead); writing – review and editing (lead).

## FUNDING INFORMATION

This work was supported by grants from the National Nature Science Foundation of China (No. 81860487; 82060216), Yunnan Health Training Project of High Level Talents (No. D2018031), and the research and teaching reform project of Kunming Medical University (2018‐JY‐Y‐042). The funders did not participate in the experimental research or the preparation of the manuscript.

## CONFLICT OF INTEREST STATEMENT

The authors declare that they have no conflicts of interest.

## ETHICS STATEMENT

This study was carried out in accordance with the “Declaration of Helsinki” and approved by the Ethics Committee of the First affiliated hospital of Kunming Medical University. The written informed consents were obtained from all individual participants included in the study. All the participants were notified that they have the right to refuse or terminate the study at any point of the interview. Animal husbandry, care, and all experiments were carried out in accordance with the Animal Research: Reporting of In Vivo Experiments (ARRIVE) guidelines, all animal experiments were approved and supervised by the Animal Care Committee of Kunming Medical University.

## Data Availability

All data generated or analyzed during this study are included in this article. Further inquiries can be directed to the corresponding author on reasonable request.
